# Tracking genetic diversity in amur tigers: a long-term study using microsatellites in Southwest Primorye, Russia

**DOI:** 10.1007/s11033-025-10339-z

**Published:** 2025-02-26

**Authors:** Jangmi Lee, Taisiia Marchenkova, Dina Matiukhina, Anya Lim, Yung Kun Kim, Daecheol Jeong, Jee Yun Hyun, Sujoo Cho, Dong Youn Kim, Ying Li, Yury Darman, Mi-Sook Min, Je-Yeol Cho, Victor Bardyuk, Younghee Lee, Puneet Pandey, Hang Lee

**Affiliations:** 1 Research Institute for Veterinary Science and Conservation Genome Resource Bank for Korean Wildlife, College of Veterinary Medicine, Seoul National University, Seoul, South Korea; 2 Tiger and Leopard Conservation Fund in Korea, Seoul, South Korea; 3 Federal State Budgetary Institution Joint Directorate of Kedrovaya Pad’ State Biosphere Nature Reserve and Land of the Leopard National Park, Ministry of Natural Resources and Environment of the Russian Federation, Vlaaffiliationostok, Primorsky Krai Russia; 4 Research Center for Endangered Species, National Institute of Ecology, Gyeongbuk, South Korea; 5 National Institute of Biological Resources, Incheon, South Korea; 6 Department of Agriculture, Forestry and Bioresources, College of Agriculture and Life Sciences, Seoul National University, Seoul, South Korea; 7 Veterinary Humanities and Social Science, College of Veterinary Medicine, Seoul National University, Seoul, South Korea; 8 College of Geography and Ocean Science, Yanbian University, Yanji, Jilin China; 9 Pacific Institute of Geography, Far Eastern Branch of Russian Academy of Science, Vlaaffiliationostok, Russia; 10 Laboratory of Veterinary Biochemistry, College of Veterinary Medicine, Seoul National University, Seoul, South Korea; 11 College of Veterinary Medicine and Research Institute for Veterinary Science, Seoul National University, Seoul, South Korea

**Keywords:** Amur tiger, Southwest primorye, Genetic diversity, Microsatellites

## Abstract

**Background:**

The tiger population in Southwest Primorye is small and predominantly isolated from the main Sikhote-Alin population, which constitutes approximately 90% of the wild Amur tiger population. By 1996, this population declined to fewer than 10 individuals, but it has since grown and expanded into nearby habitats, now numbering over 50 individuals. Therefore, the regular genetic monitoring of this population is essential, as it has grown from a few founding members and remained geographically isolated.

**Methods and results:**

Genetic diversity was assessed using nine heterologous microsatellite markers amplified from non-invasively collected samples of 20 individual tigers. The Southwest Primorye tiger population exhibited moderate genetic diversity, with allelic richness (Na) at 3.67 and observed heterozygosity (Ho) at 0.63. Additionally, we detected a slight tendency toward heterozygosity excess at several loci, with an overall negative FIS, which may be influenced by recent genetic admixture or subtle population structuring. comparative assessment between our study and Sugimoto et al. (2012) revealed a marginal increase in genetic diversity over time, suggesting improved genetic health of the population, potentially due to genetic exchange with other populations.

**Conclusions:**

The significant growth and expansion of the Southwest Primorye tiger population into adjacent areas of Northeast China over the past two decades suggest a positive population trajectory. This modest increase in genetic diversity indicates a potentially favorable population condition. However, continuous genetic monitoring remains essential to track genetic trends, detect potential risks, and inform conservation strategies. This study highlights the need for ongoing evaluations to ensure the long-term survival of the Amur tiger population in Southwest Primorye.

**Supplementary Information:**

The online version contains supplementary material available at 10.1007/s11033-025-10339-z.

## Introduction

The genetic makeup of a species plays a critical role in its long-term survival by providing resilience against disease and environmental changes, and ensuring reproductive fitness [1–3]. Small populations are particularly vulnerable to the detrimental effects of inbreeding, which can lead to an increased risk of decline and eventual extinction [4]. Conservation strategies must focus on preserving and enhancing genetic diversity through measures such as habitat protection, breeding programs, and genetic rescue operations. These efforts help maintain the adaptive potential of species, enabling them to evolve in response to changing conditions and ensuring their continued existence. Without careful genetic management, the long-term survival of endangered species remains in jeopardy.

The Amur tiger, also known as the Siberian tiger (*Panthera tigris altaica*), is one of the five remaining tiger subspecies and serves as a keystone and umbrella species in East Asian ecosystems [5–7]. Genetic study has revealed that the extinct Korean tigers were part of the Amur tiger lineage [8]. Like other tiger subspecies, the Amur tiger has experienced significant population decline and range collapse due to habitat loss, illegal hunting, and capture for economic gain [9, 10]. By the mid-20th century, it was estimated that approximately 150 tigers remained in Manchuria, with no more than 50 individuals in the Russian Far East [11, 12]. Currently, the population in Russia is estimated at about 750 individuals (including cubs) [13] and split into two groups: a larger group in the Sikhote-Alin Mountains and a smaller one in Southwest Primorye [14].

**Fig. 1:**
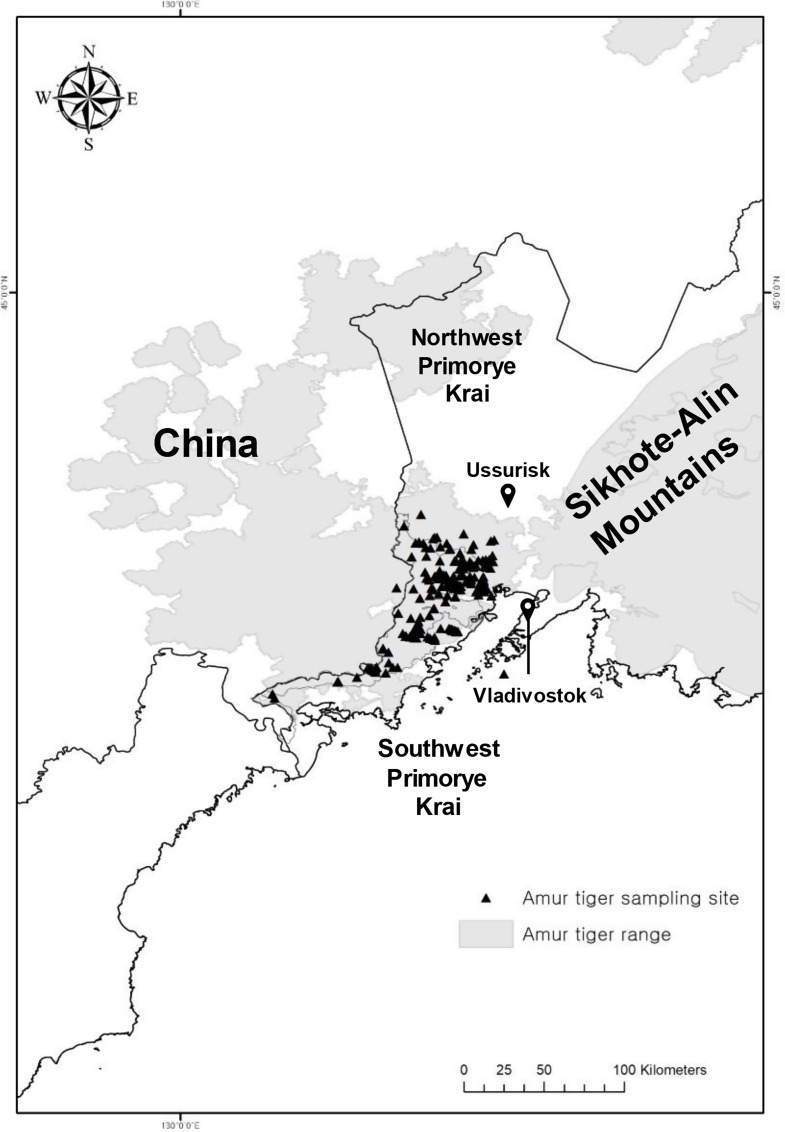
Amur tiger sampling sites and distribution

Despite its small size, the Southwest Primorye population of Amur tigers holds high conservation importance. Its geographical proximity to China and the Korean Peninsula makes it crucial for the revival of tigers in these historical distribution areas [15] (Fig. 1). This population is predominantly isolated from the larger Sikhote-Alin population due to human development between Vladivostok and Ussuriysk, which presents a barrier to tiger dispersal and gene flow [16]. However, some individuals have managed to migrate between regions, indicating that the barrier is not entirely impassable [17]. In 2005, the Russian-Chinese East Manchurian Amur tiger sub-population was estimated to include no more than 20 animals in total [18]. By 2015, this number had grown to 35 adults [19], and has continued to increase since. The gradual growth of this population, with more frequent tiger sightings on the Chinese side, is largely attributed to effective conservation measures and governmental policies [20–22].

The small population size and limited genetic exchange with the larger Sikhote-Alin population necessitate periodic genetic management of the Southwest Primorye tiger population to ensure its long-term viability, growth, and expansion. Despite this critical need, genetic studies on the Southwest Primorye population are limited due to its transnational distribution (Russia-China) and the lack of a universally accepted genetic methodology for global tiger studies. Between 2000 and 2019, three different studies monitored the Southwest Primorye tiger population using noninvasive samples and microsatellites over the periods of 2000–2004 [23], 2012–2016 [13], and 2014–2019 [24]. Sugimoto et al. [23] and Ning et al. [13] used heterologous microsatellites derived from domestic cats, while Jeong et al. [24] employed tiger-specific genomic microsatellite markers developed by Hyun et al. [25]. Although the observed (Ho) and expected (He) heterozygosity were consistent across all three studies, averaging around 0.6, the mean allelic richness varied, with values of 3.2 [23], 3.7 [13], and 2.6 [24]. As each study employed a different set of microsatellites, cross-study comparisons are challenging, making it difficult to determine whether this population has experienced an increase, decrease, or maintenance of genetic diversity over time.

The present study was designed to monitor potential fluctuations in genetic diversity over time in the tiger population of Southwest Primorye in the Russian Far East. We amplified nine heterologous microsatellite loci using DNA from 20 tiger individuals sampled non-invasively between 2014 and 2019 [24]. The findings were then compared with those of Sugimoto et al. [23], who sampled tigers from the same study area between 2000 and 2004, enabling a comparative assessment over approximately 15 years, a period spanning more than two tiger generations.

## Methodology

Between 2014 and 2019, Jeong et al. [24] non-invasively sampled the tiger population in the Land of the Leopard, located at the southern tip of Primorsky Province in the Russian Far East. This area includes two specially protected areas: the Kedrovaya Pad Nature Reserve and the Land of the Leopard National Park. They identified 32 unique tiger individuals using ten polymorphic microsatellite loci. In the present study, we utilized DNA from 20 of these 32 tigers to amplify nine heterologous microsatellites. As no additional sampling efforts were required, no new permissions, permits, or ethical clearances were needed.

The nine polymorphic heterologous microsatellites identified by Sugimoto et al. [23] were amplified using the M13-tailed primer method. In this cost-effective approach, the forward primer for each microsatellite primer set was designed with a 5′-tail containing the M13 sequence 5′-CACGACGTTGTAAAACGAC-3′ [26, 27]. A third M13 primer, which carried a fluorescent label, bound to the forward primer during PCR amplification. Given that previous studies [13, 23, 24] used fluorescent dye-labeled microsatellite loci, we additionally fluorescently labeled two of the nine heterologous microsatellite markers (FCA94 and FCA224) and amplified them in a subset of samples to assess the impact of the M13 tailing method on our results.

The PCR reactions were performed in a 10 µL mixture composed of 5 µL multiplex master mix (QIAGEN Multiplex PCR Kit), 0.5 µL Q solution (QIAGEN, Hilden, Germany), 1.0 µL (10 pmol) primer mix, and 2 µL template DNA. The PCR amplifications were checked on a 2% agarose gel, and positive amplifications were genotyped on a 3730XL genetic analyzer at NICEM Inc. (Seoul, South Korea) with a Genescan 500 LIZ internal size standard. Data calling was performed using GeneMapper v3.7 (Applied Biosystems, Foster City, CA).

To construct the genotypic profile, a multiple-tube approach was used for each sample. Initial triplicate PCR assays were conducted, followed by an additional 2–3 PCR assays for samples that failed to produce a consensus genotype in the initial step. The consensus genotype was confirmed under the following criteria: genotypes were accepted when triplicates matched for homozygotes, or when at least two replicates matched for heterozygotes.

Micro-Checker version 2.2.3 [28] was used to detect the presence of null alleles. Hardy-Weinberg equilibrium (HWE) and linkage disequilibrium (LD) were assessed using Genepop version 4.7.5 [29] with Bonferroni correction applied [30]. The amplification success rate (ASR), defined as the ratio of successful amplifications to the total number of PCR attempts, and the genotyping success rate (GSR), defined as the ratio of observed consensus profiles to the total number of samples analyzed, were calculated for each amplified microsatellite locus. Genotyping errors, including allele dropout and false alleles, were assessed using GIMLET version 1.3.3 [31]. Genetic diversity parameters, such as allele number and heterozygosity, were calculated using CERVUS version 3.0.7 [32]. Inbreeding within the population was assessed using FSTAT version 2.9.4 [33], while individual-level inbreeding was analyzed with Coancestry version 1.0.1.11 [34] The Factorial Correspondence Analysis (FCA) was conducted based on allele frequencies to visualize genetic structure and identify population clustering [35].

## Results

Eight of the nine loci were tested across all 20 samples, except for FCA94, which was tested with only five samples due to DNA depletion in the remaining samples during the experiments. The nine heterologous microsatellite markers amplified exhibited no deviation from Hardy-Weinberg equilibrium (HWE), and there were no signs of null alleles or linkage disequilibrium (LD) after Bonferroni correction. The microsatellite markers showed an average amplification success rate of 65.4% and a genotyping success rate of 95% (Supplementary Table 1). The allele dropout rate per locus ranged from 7 to 33%, with a mean of 19%, while the false allele rate per locus ranged from 0 to 8%, with a mean of 3% (Table 1). The number of alleles ranged from two to five per locus, averaging 3.7 per locus (Table 1). The observed heterozygosity ranged from 0.47 to 0.85 (mean 0.63 per locus), while the expected heterozygosity ranged from 0.49 to 0.77 (mean 0.63 per locus).

**Table 1:**
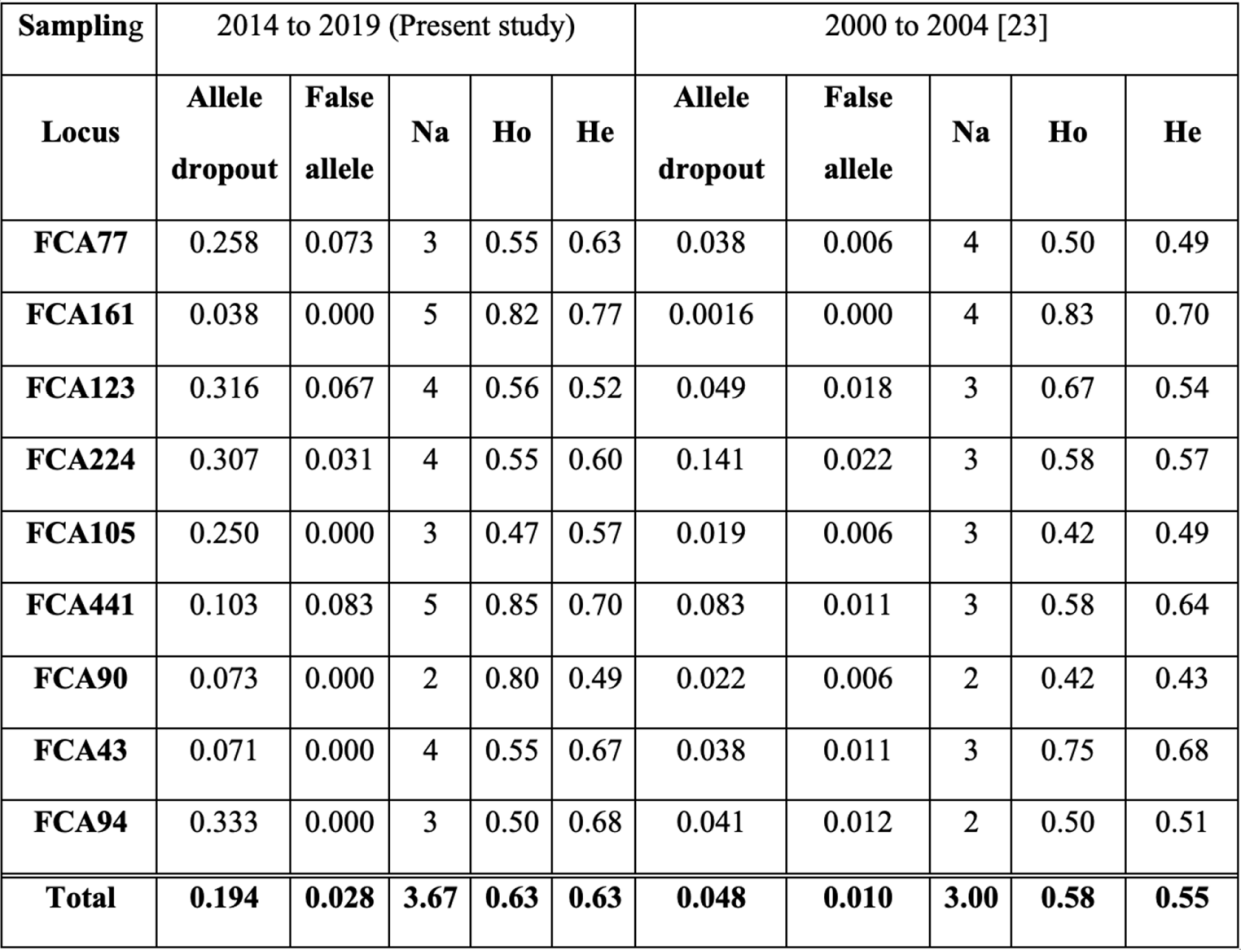
Genotyping errors and genetic diversity of the Southwest Primorye tiger population using noninvasive samples collected between 2000 and 2019 (na: number of alleles; Ho: observed heterozygosity; he: expected heterozygosity)

**Fig. 2:**
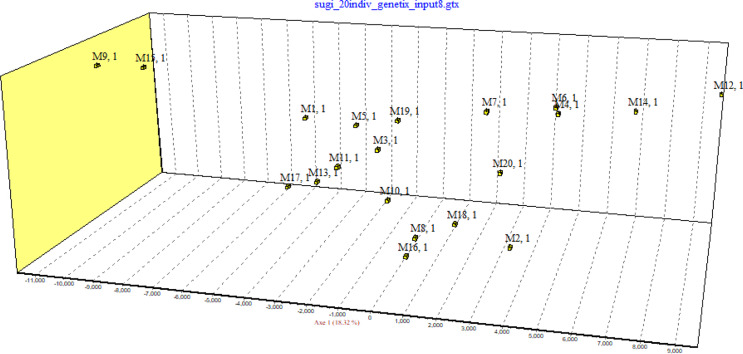
Factorial correspondence analysis of genetic variation in 20 Amur tiger individuals

Inbreeding coefficients were calculated using microsatellite data from eight loci, excluding FCA94, which was analyzed in only five samples. The overall mean F_IS_ across all loci was slightly negative (-0.05575), ranging from − 0.652 at FCA90 to 0.179 at FCA105 and FCA43 (Supplementary Table 2). The mean inbreeding coefficients across individuals were slightly negative for both Ritland (-0.0326) and LynchRd (-0.0408), while the mean values for TrioML (0.0957) and DyadML (0.1091) were positive (Supplementary Table 3). These findings suggest a genetically diverse population with minimal inbreeding effects. Additionally, Factorial Correspondence Analysis (FCA) revealed spatial differentiation of individuals along the primary axis, suggesting potential genetic structuring. This pattern is characterized by the formation of closely associated clusters among some individuals, while others exhibit a more dispersed distribution (Fig. 2).

The genetic diversity estimates obtained in this study from tiger samples collected between 2014 and 2019 were higher than those reported for samples collected between 2000 and 2004 [23]. Specifically, from 2000 to 2019, the mean allelic diversity increased from 3 to 3.7 alleles per locus. Similarly, the mean observed heterozygosity rose from 0.59 to 0.63, and the mean expected heterozygosity from 0.55 to 0.63 per locus. Interestingly, the mean genotyping error rates reported in our study (ADO – 0.19, FA – 0.03) were also higher than those previously reported (ADO – 0.05, FA – 0.01; [23]) using the same set of microsatellites and non-invasive samples. The higher genotyping error rates may have resulted from relatively poor quality of the extracted DNA or from the use of M13-tailed primers. The mean PCR amplification success rate in our study was 65.4%, which is lower than the rates reported by Jeong et al. (2024), who achieved 84.8% for the ten polymorphic microsatellites used for individual identification and 89.4% for all 32 microsatellite loci tested during the initial screening. We further conducted a comparative assessment of M13-tailed and fluorescently labeled microsatellites by amplifying two loci (FCA 94 and FCA 224) in a sample subset. The results clearly suggest that M13-tailed primers have lower amplification success rates (FCA 94–79%, FCA 224–28%; Table 2) compared to fluorescently labeled primers (FCA 94–100%, FCA 224–64%; Table 2). These observations strongly suggest that the use of M13-tailed primers, rather than DNA quality, influenced the genotyping error rate. However, we were unable to draw definitive conclusions about the comparative error rates between primers, as locus FCA 94 exhibited higher allele dropout with the fluorescently labeled primer, but locus FCA 224 showed higher dropout with the M13-tailed primer (Table 2).

**Table 2:**
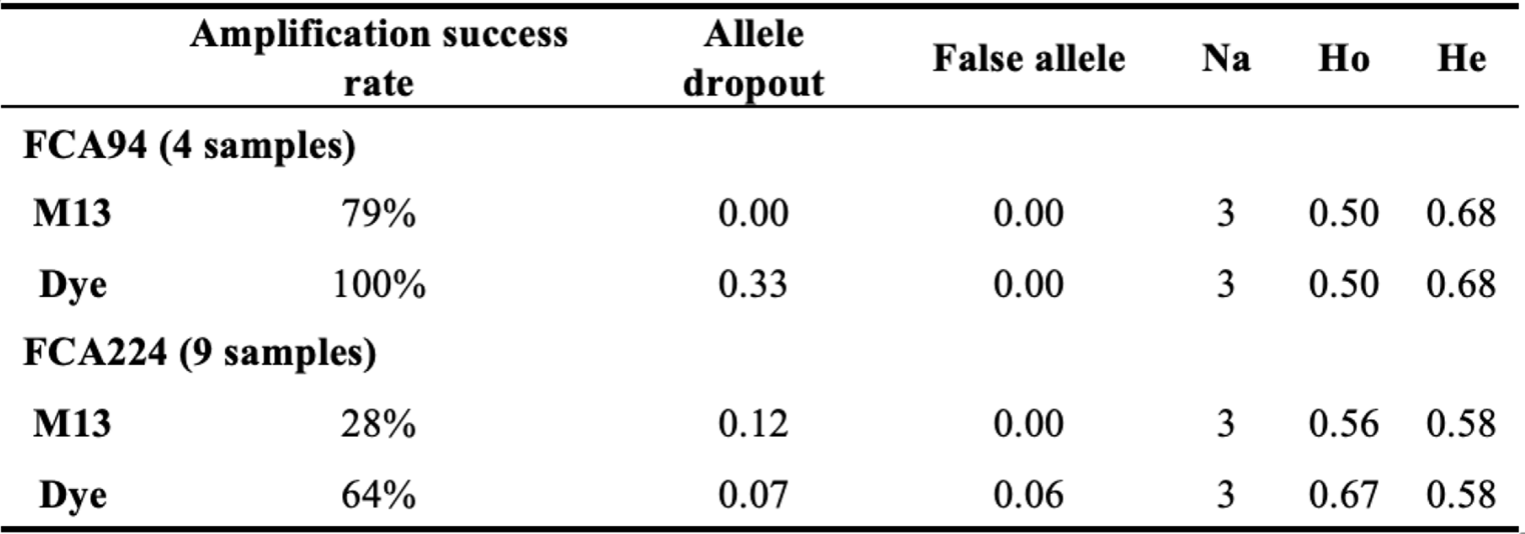
Comparative assessment of M13-tailed and dye-labeled microsatellite amplification

## Discussion

Over the past few decades, the tiger population in Southwest Primorye has been gradually increasing due to improved management strategies and enhanced landscape conservation efforts. In 2022, snow footprint tracking identified 58 tigers, including 12 cubs, within the Southwest Primorye territory- an increase from 13 animals in 2005 and 27 in 2015 [36]. This rise in tiger numbers has facilitated the movement of tigers to nearby former territories in China, where more than 50 tigers are now distributed across the Laoyeling, Zhangguangcailing, Wandashan, and Lesser Khingan Mountains [37].

Effective genetic management is crucial for expanding populations, particularly those originating from a small initial group, as they are more vulnerable to fluctuations in genetic diversity due to factors such as the founder effect, genetic drift, population bottlenecks, selection pressure, inbreeding, gene flow, and the formation of sub-populations. Our comparative genetic assessment, spanning approximately 15 years and more than two tiger generations, provides valuable insight into how population growth, territorial expansion, and conservation measures have influenced genetic diversity in Southwest Primorye. Given the average lifespan and breeding patterns of tigers, we do not expect any individual recaptures between this study and the prior one.

Our findings reveal a moderate level of genetic diversity in the Southwest Primorye tiger population, with comparative analysis suggesting a slight increase between 2000 and 2019 (Table 1). This marginal rise in genetic diversity may be attributed to gene flow from nearby populations or an improved ability to avoid inbreeding as the population expands. The former is more likely, given the overall negative FIS and subtle sub-structuring. Furthermore, our observations align with previous genetic studies. Sorokin et al. (2016) reported genetic exchange between the Southwest Primorye population and the main population in the Sikhote-Alin Mountains. Additionally, Jeong et al. (2024) identified two haplotypes (Type A and Type G) in Southwest Primorye, with Type G individuals being geographically restricted to the northern part of the study area.

In this study, we used M13-tailed microsatellite loci due to cost constraints, as this method is more cost-effective compared to labeling each microsatellite forward primer with fluorescent dye. However, we observed a reduced PCR amplification success rate compared to Jeong et al. (2024), despite obtaining DNA from the same samples. Additionally, we reported a higher mean genotyping error rate for the tested microsatellite loci compared to Sugimoto et al. (2012). The use of M13 as a label has been associated with decreased PCR efficiency, often necessitating additional amplification cycles [38]. Furthermore, extensive homology of the M13 sequence and the target genome can lead tononspecific amplifications, potentially introducing errors in genotyping analysis [39]. Therefore, future studies should either avoid the use of M13-tailed primers or apply with caution when necessary.

## Conclusion

Conserving the genetic diversity of the Southwest Primorye Amur tiger population is vital for its long-term viability and potential southward range expansion. The Southwest Primorye population is relatively small and largely isolated from the main Sikhote-Alin tiger population, which represents nearly 90% of all Amur tigers, due to barriers created by human development. Given the small and isolated nature of this population, periodic studies are essential to evaluate trends in genetic diversity and monitor changes over time. Such data is critical for developing informed conservation strategies, including habitat connectivity and potential genetic rescue efforts. By prioritizing advanced genetic tools, we can better understand the genetic dynamics of the Amur tiger population, mitigate risks associated with genetic isolation, and enhance the effectiveness of conservation initiatives aimed at ensuring the survival of this iconic species in its natural habitat.

## Electronic supplementary material

Below is the link to the electronic supplementary material.

Supplementary Material 1

## Data Availability

No datasets were generated or analysed during the current study.
