# Supplementary material for: Tracking genetic diversity in amur tigers: a long-term study using microsatellites in Southwest Primorye, Russia
Source: Mol Biol Rep. 2025 Feb 26;52(1):264. doi: 10.1007/s11033-025-10339-z (PMC11865129; doi:10.1007/s11033-025-10339-z)
Supplement: Supplementary file 1 — Supplementary Material 1 [file 11033_2025_10339_MOESM1_ESM.docx]

**Supplementary Table 1. Amplification and genotyping success for nine heterologous microsatellite markers**

**
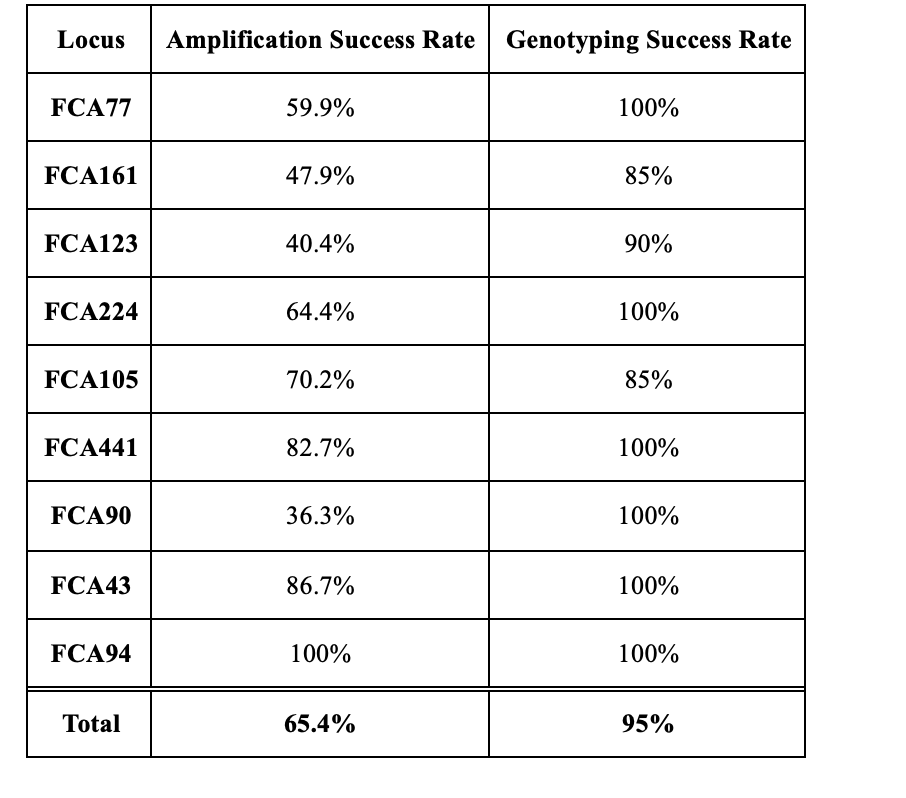
**

**Supplementary Table 2. Population-level inbreeding coefficients (F_IS_)**

| F_IS_ per population | |
| --- | --- |
| FCA77 | 0.126 |
| FCA161 | -0.067 |
| FCA123 | -0.073 |
| FCA224 | 0.083 |
| FCA105 | 0.179 |
| FCA441 | -0.221 |
| FCA90 | -0.652 |
| FCA43 | 0.179 |
| Total | -0.05575 |

**Supplementary Table 3. Individual inbreeding coefficients by estimator**

| Individual | Ritland | LynchRd | TrioML | DyadML |
| --- | --- | --- | --- | --- |
| 1 | -0.281 | -0.3372 | 0 | 0 |
| 2 | 0.0314 | 0.0487 | 0.143 | 0.1544 |
| 3 | -0.1409 | -0.0914 | 0.0061 | 0.0075 |
| 4 | -0.2389 | -0.351 | 0.003 | 0.0026 |
| 5 | -0.0632 | -0.0824 | 0.0193 | 0.0225 |
| 6 | 0.0502 | 0.0371 | 0.1536 | 0.1727 |
| 7 | 0.185 | 0.3304 | 0.3603 | 0.3886 |
| 8 | -0.0524 | -0.0998 | 0.0164 | 0.0148 |
| 9 | 0.3645 | 0.2882 | 0.3781 | 0.4043 |
| 10 | -0.2182 | -0.2268 | 0.0001 | 0.0001 |
| 11 | -0.1281 | -0.0813 | 0.0149 | 0.0292 |
| 12 | 0.6192 | 0.4844 | 0.305 | 0.3457 |
| 13 | -0.0828 | -0.1534 | 0.0324 | 0.0535 |
| 14 | -0.1652 | -0.161 | 0.0055 | 0.0219 |
| 15 | -0.1095 | -0.0558 | 0.0241 | 0.0341 |
| 16 | 0.2519 | 0.411 | 0.2924 | 0.3414 |
| 17 | -0.2879 | -0.228 | 0 | 0 |
| 18 | 0.0314 | 0.0432 | 0.1462 | 0.1813 |
| 19 | -0.2626 | -0.3565 | 0.0019 | 0 |
| 20 | -0.1556 | -0.2348 | 0.0108 | 0.0067 |
| Total | -0.0326 | -0.0408 | 0.095655 | 0.109065 |
